# An isolated vaginal metastasis from rectal cancer: a case report

**DOI:** 10.1186/s13256-024-04501-7

**Published:** 2024-05-02

**Authors:** Saida Sakhri, Ines Zemni, Mohamed Ali Ayadi, Ayoub Ghazouani, Nadia Boujelbene, Tarek Ben Dhiab

**Affiliations:** 1 Department of Surgical Oncology, Faculty of Medicine of Tunis, Salah Azaiez Institute, University of Tunis El Manar, Boulevard 9 Avril 1938, Tunis, Tunisia; 2 LMBA (LR03ES03), Sciences Faculty of Tunis, University Tunis el Manar, Tunis, Tunisia; 3 Department of Pathology, Faculty of Medicine, LMBA (LR03ES03), Sciences Faculty of Tunis, Salah Azaïz Institute, University Tunis El Manar, Tunis, Tunisia

**Keywords:** Vaginal cancer, Colorectal metastasis, Oligometastatic disease

## Abstract

**Introduction:**

Vaginal metastasis from colorectal cancer is a rare occurrence, typically associated with other metastatic lesions. Isolated metastasis is exceedingly uncommon, with only a few cases documented in the literature. Vaginal involvement in colorectal cancer primarily results from direct contiguous spread from the primary tumor.

**Case presentation:**

We present the case of a 70-year-old African woman diagnosed with adenocarcinoma of the middle rectum. She underwent chemotherapy, radiotherapy, and subsequent anterior resection. After 2 months, an isolated metastasis of rectal cancer was identified in the lower third of the left vaginal wall, confirmed by biopsy. Colonoscopy ruled out colorectal recurrence. Thoraco-abdominal computed tomography scan showed no distant metastases. The patient underwent abdominoperineal resection, removing the lateral and posterior vaginal wall with free macroscopic margins and a definitive colostomy. The final histopathological analysis confirmed the diagnosis of moderately differentiated adenocarcinoma of the vagina, measuring 5 × 4.5 cm. The rectal wall was extrinsically invaded by the tumor down to the muscularis propria while respecting the rectal mucosa. Resection margins were negative. The patient was discharged 1 week postoperation with no complications. Adjuvant chemotherapy was indicated, and the patient is currently tolerating the treatment well.

**Conclusion:**

Vaginal metastases from colorectal cancer are extremely rare. A vigilant gynecological examination is recommended during the follow-up of colorectal cancer patients. Diagnosis can be challenging, especially if the metastatic lesion is small and asymptomatic, even after standard radiological examination. Surgical resection followed by chemotherapy is a valid option for patients with early isolated metastases.

## Introduction

Colorectal cancer is one of the most common digestive cancers, with liver and lungs being the primary sites of metastasis. Vaginal metastasis is a rare phenomenon, usually occurring alongside other metastatic lesions. Isolated metastases are exceptionally rare, with the first reported case by Whitelaw in 1956. Vaginal involvement in colorectal cancer is mostly owing to direct contiguous spread from the primary tumor [2, 3].

This article aimed to review the clinical presentation, treatment, and prognosis of vaginal metastases from colorectal malignancy on the basis of existing literature.

## Case presentation

We present the case of a 70-year-old African woman with no family history, who underwent hystero-oophorectomy for a uterine myoma 30 years ago. She was referred to our institute owing to perineal discomfort and abdominal fullness associated with minimal rectal bleeding over 2 months. Physical examination revealed a soft, lax abdomen with no lymphadenopathy. Digital examination discovered an irregular posterior rectal tumor located 7 cm from the anal margin. Gynecological examination was normal, and the rectovaginal septum was intact.

Colonoscopy revealed an ulcerated tumor with a sharply demarcated margin, 4 cm high, hemi-circumferential posterior to the middle rectum. Initial laboratory results showed a carcinoembryonic antigen level of 6.56 ng/ml. Biopsy confirmed a diagnosis of well-differentiated rectal adenocarcinoma. Magnetic resonance imaging (MRI) displayed significant thickening of the middle rectum wall, extending 45 cm with a T2 diffusion hyper signal. The tumor, staged T3N1MX, exhibited infiltration of perirectal fat and multiple enlarged lymph nodes within perirectal and presacral spaces.

**Fig. 1:**
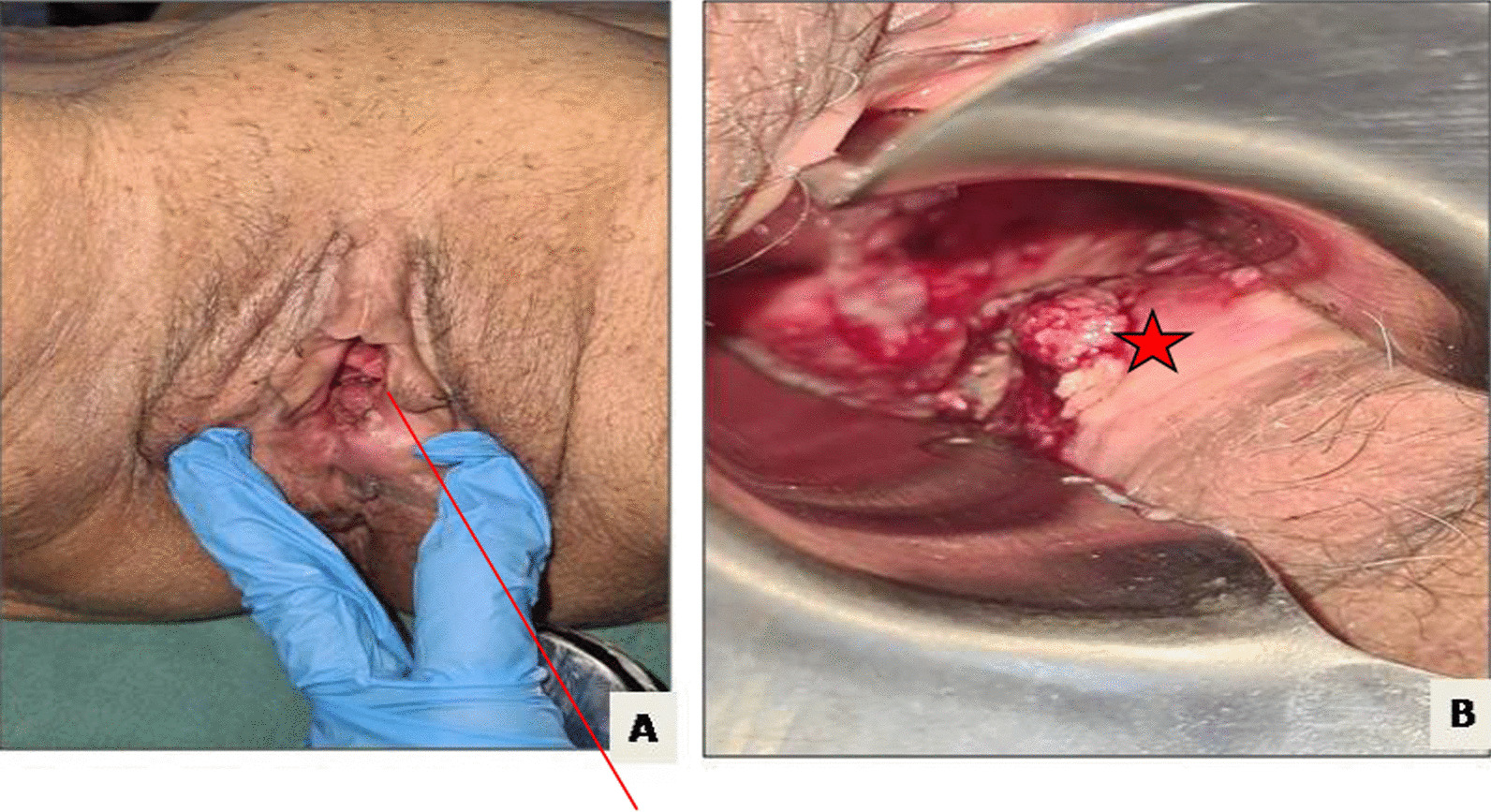
Gynecological examination showing the tumor in the entrance of the vagina marked by red line (**A**) and star (**B**)

**Fig. 2:**
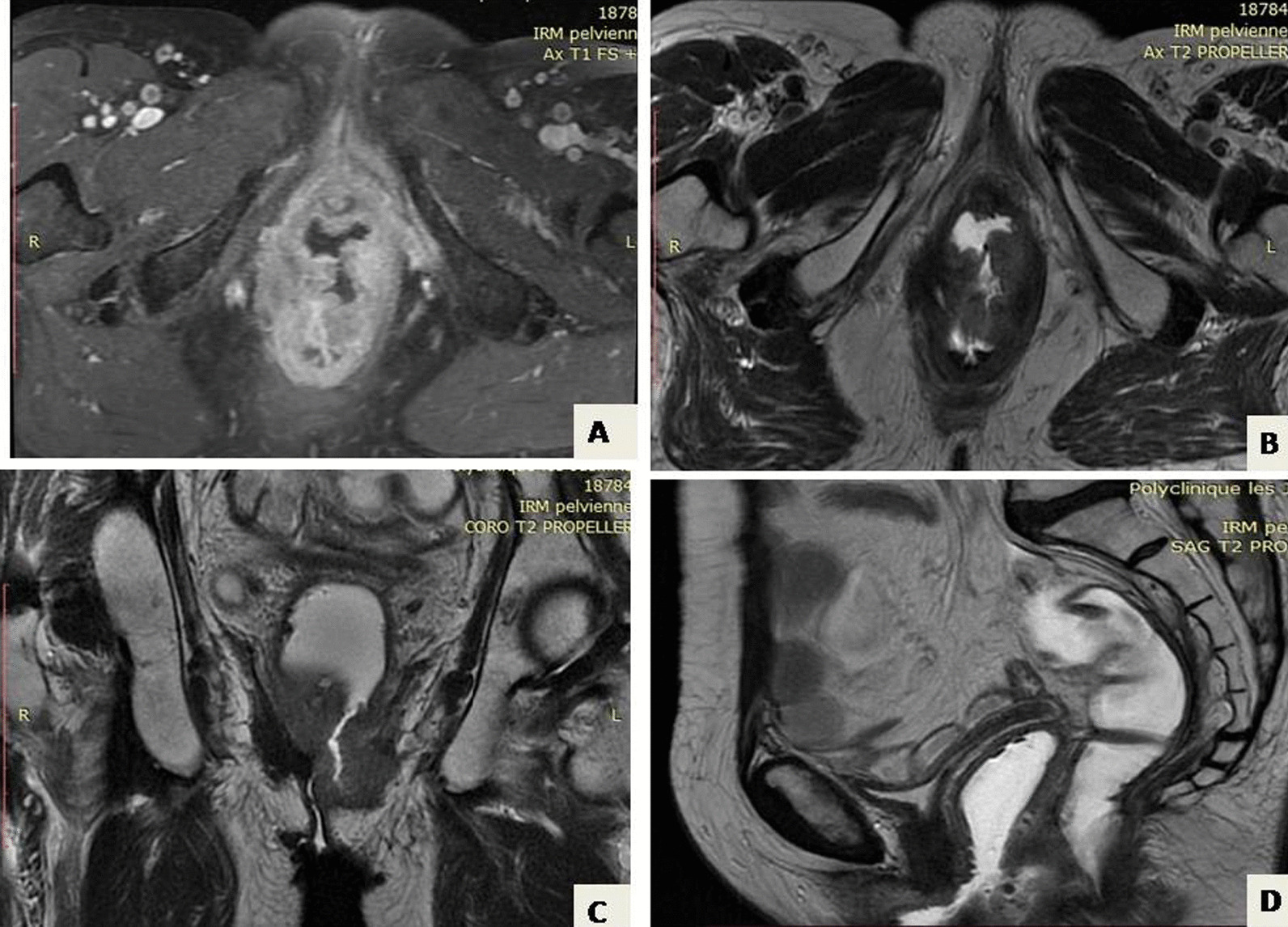
Pelvic MRI shows the vaginal tumor located in the posterior left wall of the vagina invading extrinsically into the rectum. **A** T1-weighted axial image shows the vaginal tumor. **B** T2-weighted axial image. **C** T2-weighted coronal image. **D** Sagittal view

**Fig. 3:**
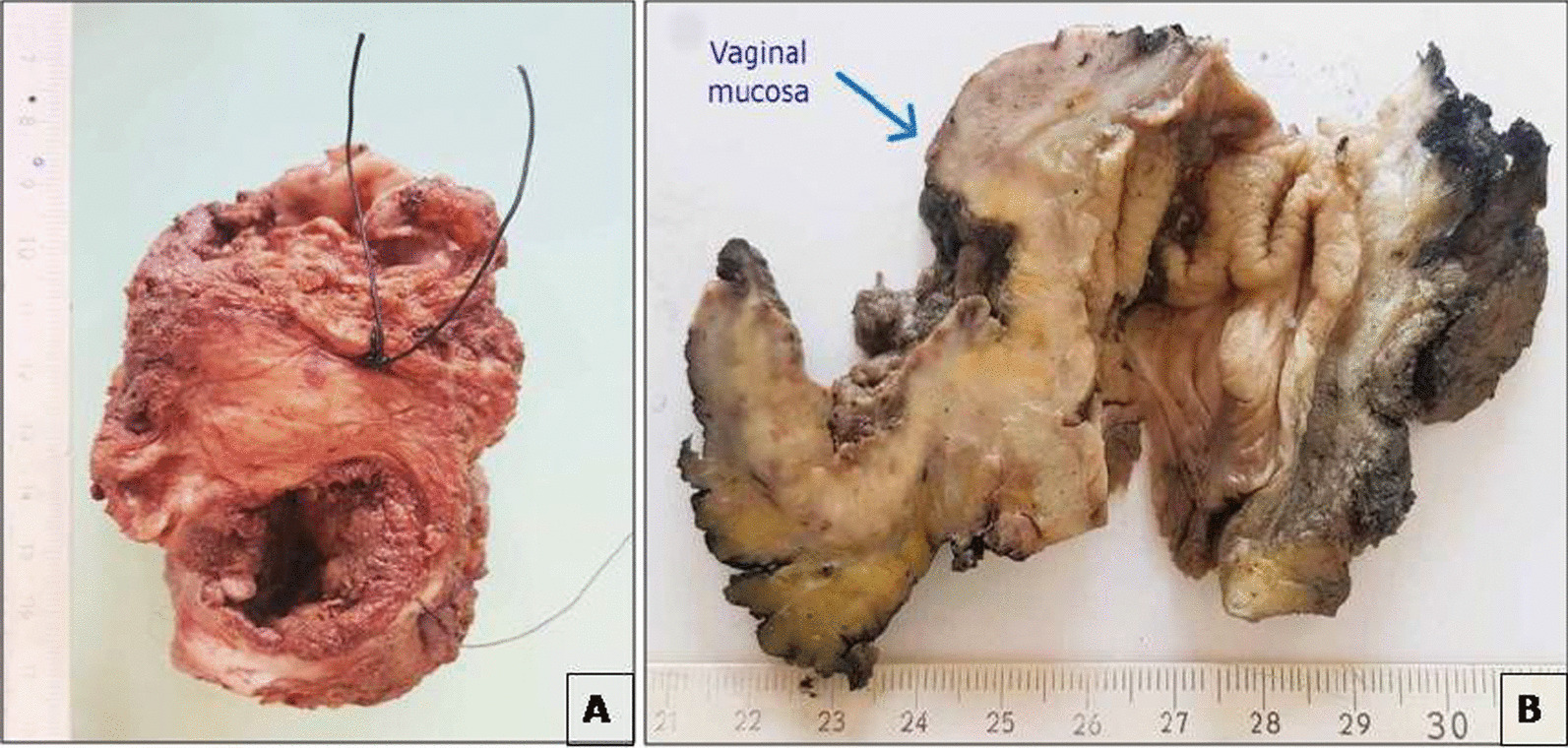
**A** Ulcerated and perforated vaginal mucosa owing to a rectal tumor process. **B** Macroscopic cross-section of the specimen

**Fig. 4:**
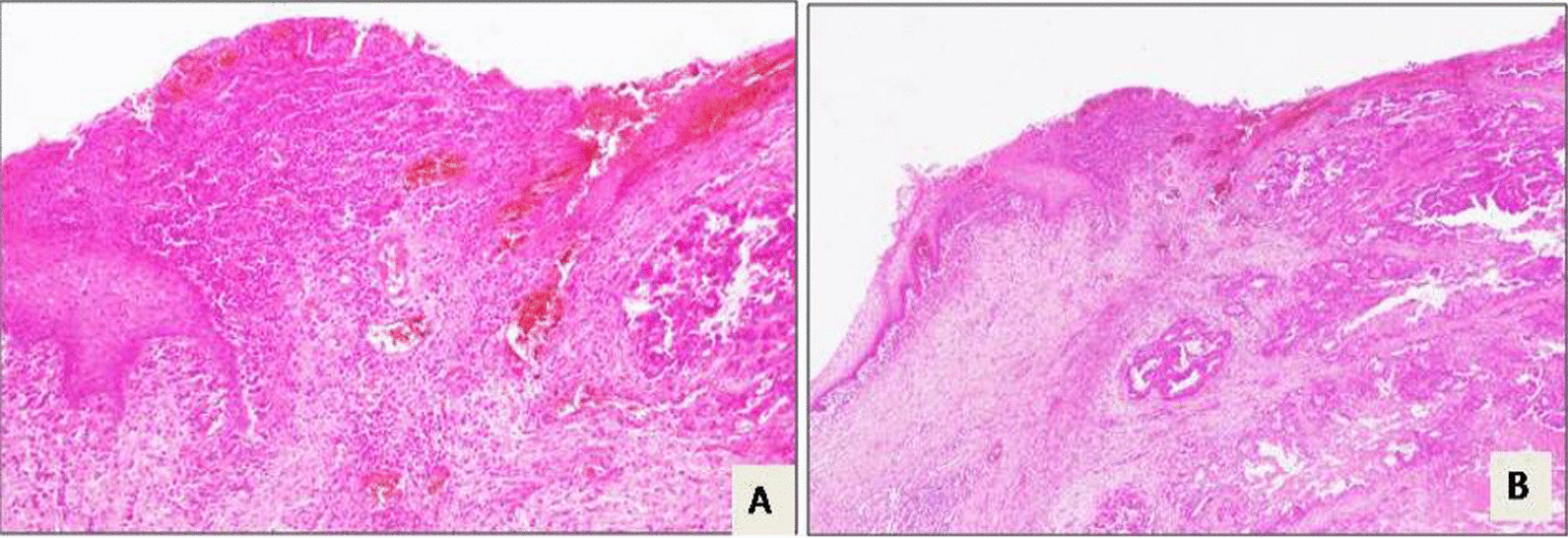
Rectal carcinoma that invades and causes ulcers in the vaginal mucosa. **A** Hematoxylin-and-eosin-stained sample under 4× magnification. **B** Hematoxylin-and-eosin-stained sample under 100× magnification

Neoadjuvant radiochemotherapy was decided upon in a multidisciplinary committee, and post-therapy evaluation indicated tumor regression. A subsequent laparotomy included anterior resection, total mesorectum excision, colorectal anastomosis, and temporary ileostomy. Histopathological analysis confirmed moderately differentiated adenocarcinoma with negative resection margins (R0) and ypT3N1 staging. Adjuvant chemotherapy followed, with ileostomy closure 1 month later. After 2 months, the patient reported lower abdominal discomfort and occasional vaginal spotting. Examination revealed a palpable vaginal mass, with invasion of the rectovaginal septum but a tumor-free cervix (Fig. 1). Biopsy confirmed colic adenocarcinoma in the vagina both morphologically and immunohistochemically (positive for CDX2 and negative for PAX8). Pelvic MRI displayed a 4 cm vaginal tumor, hyper-intense on T2-weighted images, invading the rectovaginal septum but not the colorectal anastomosis (Fig. 2). Surgery was recommended, leading to abdominoperineal resection, removing the lateral and posterior vaginal wall with free margins, and a permanent colostomy. The final histopathological analysis of the resected specimen confirmed the diagnosis of a moderately differentiated adenocarcinoma in the vagina, measuring 5 × 4.5 cm. The rectal wall was extrinsically invaded by the tumor, extending up to the muscle propria. The resection margins were negative (Figs. 3, 4). Adjuvant chemotherapy is ongoing with good tolerance.

## Discussion

Primary vaginal cancer is rare, comprising about 2% of gynecologic malignancies. Metastasis to the vagina, especially from colorectal cancer, is even rarer. When colorectal cancer metastasizes to the genital tract the most frequent site is the ovaries followed by the vagina and endometrium [2]. Four mechanisms of colorectal cancer spread to the vagina have been proposed. The first involves local invasion through direct extension, where the tumor spreads from the ischiorectal fossa and the junction of the deep and superficial perineal membranes. The second mechanism is through the transcoelomic route, wherein tumor cells invade the fallopian tube or uterus before reaching the vagina. The third mechanism is via the lymphatic system, with metastatic cells occurring in the iliac and hypogastric lymph nodes before spreading to the periurethral area and anterior vaginal wall. The final hypothesis suggests a hematogenous route, where metastatic cells travel from the primary tumor to the ovarian plexus and then through the vaginal veins. This last hypothesis appears to be more acceptable for differentiating vaginal metastases from colorectal cancer [1, 2]. In the current case, this hypothesis also appears to be the most plausible, particularly considering that the tumor invaded the rectum extrinsically, while the mucosa remained free of the tumor.

Another mechanism that was reported, as per the study by Gündoğan *et al*., involves local dissemination following transvaginal specimen extraction during laparoscopic surgery for colon cancer [4]. The average age at the time of presentation was 60.6 years. The most common clinical presentation was vaginal bleeding, which is similar to the signs of primary vaginal cancer. Other symptoms included vaginal discharge, staining, or a palpable mass [2, 5].

The diagnosis is established through clinical signs and gynecological examination, with confirmation achieved through biopsy of the lesion. Radiological examination, specifically magnetic resonance imaging (MRI), can be helpful in detecting vaginal metastasis [1]. F-fluorodeoxyglucose positron emission tomography (FDG PET)/computed tomography (CT) is useful for the diagnosis of vaginal metastasis at an early stage and to detect distant metastasis [6].

As vaginal metastases are viewed as an intermediate stage between locoregionally confined disease and metastases, a definitive treatment plan is not yet firmly established. In the literature, there is no established standard treatment for vaginal metastases, making it a challenging aspect in medical practice. Consequently, there is a lack of consensus regarding the surgical treatment approach, as well as the utilization of chemotherapy and radiotherapy [2]. The cornerstone of treatment is surgical excision of vaginal mass or colpectomy; other treatment options are possible, including external beam therapy or interstitial brachytherapy. Chemotherapy is used in cases of multiple metastases. However, surgical excision remains an appropriate approach for local control [2]. In the series by Kwon *et al*., vaginal metastasis occurred 10 months after the primary surgery. It was treated similarly to primary vaginal carcinomas, employing radiotherapy followed by surgical resection. Subsequently, the patient received adjuvant chemotherapy, either individually or in combination [1, 2].

In the present case, recurrence manifested early, occurring only 2 months after the completion of treatment for the primary tumor. The multidisciplinary committee at our institute decided to proceed with surgery since the CT scan revealed no evidence of distant metastasis, and the metastasis located in the vagina was deemed resectable.

Similar to the approach taken in the study by Ansari *et al*., a patient with oligometastatic vaginal involvement of colon cancer was treated as if it were primary vaginal cancer. The patient underwent partial colpectomy, followed by pelvic radiotherapy involving inguinal lymph nodes, along with concurrent 5-fluorouracil (5-FU). Subsequently, vaginal brachytherapy was administered. This therapeutic approach was explained by the fact that the vaginal disease was situated in the distal vagina [3].

In Jene Ng’s literature review, which encompassed 37 case reports, only 9 cases presented with isolated vaginal metastasis. The primary location of the tumor in all cases was the sigmoid and rectum, and the histological type was consistently adenocarcinoma. Notably, there was no standardized treatment across the reported cases. Surgical resection was the sole treatment for one patient, while another patient received exclusive radiotherapy owing to the disappearance of the vaginal metastasis [2]. Fade Alawneh suggested an aggressive approach that involves surgical procedures such as hysterectomy and bilateral salpingo-oophorectomy, colpectomy, and a combination of radiotherapy and chemotherapy [7].

For a significant period, vaginal metastasis from colorectal cancer has been regarded as having a poor prognosis. However, current trends indicate that the adoption of aggressive approaches in both diagnosis and treatment can yield benefits, leading to improved outcomes and long-term survival [2].

## Conclusion

Vaginal metastases from colorectal cancer are exceedingly rare. It is advisable to include a thorough gynecological examination in the follow-up protocol for patients with colorectal cancer. Diagnosis can pose challenges, especially if the metastatic lesion is small and asymptomatic, even after standard radiological examination. In cases of early isolated metastases, surgical resection followed by chemotherapy emerges as a valid and effective treatment option.

## Data Availability

Data supporting our findings were taken from the patient’s folders.
